# The Characterization of Non-oncologic Chronic Drug Therapy in Bladder Cancer Patients and the Impact on Recurrence-Free and Cancer-Specific Survival: A Prospective Study

**DOI:** 10.3390/jcm12216749

**Published:** 2023-10-25

**Authors:** Dorothea Strobach, Lisa Haimerl, Hanna Mannell, Christian G. Stief, Alexander Karl, Tobias Grimm, Alexander Buchner

**Affiliations:** 1 Hospital Pharmacy and Doctoral Programm Clinical Pharmacy, LMU University Hospital, Marchioninistraße 15, 81377 Munich, Germany; lisa.haimerl@med.uni-muenchen.de; 2 Physiology, Institute for Theoretical Medicine, Faculty of Medicine, University of Augsburg, Universitätsstraße 2, 86159 Augsburg, Germany; hanna.mannell@med.uni-augsburg.de; 3 Department of Urology, LMU University Hospital, Marchioninistraße 15, 81377 Munich, Germany; christian.stief@med.uni-muenchen.de (C.G.S.); alexander.buchner@med.uni-muenchen.de (A.B.); 4 Department of Urology, Hospital Barmherzige Brüder, Romanstraße 93, 80639 Munich, Germany; alexander.karl@barmherzige-muenchen.de; 5 Urology Practice Kaufbeuren, Gutenbergstraße 8, 87600 Kaufbeuren, Germany; grimm@urologie-kaufbeuren.de

**Keywords:** bladder cancer, cystectomy, medication, polypharmacy, survival, urothelial carcinoma

## Abstract

We aimed to characterize non-oncologic chronic drug therapy of bladder cancer (BC) patients and evaluate a possible impact on recurrence-free (RFS) and cancer-specific survival (CSS). Patients with a first diagnosis (FD) of BC or radical cystectomy (RC) were included in a prospective, monocentric, observational study. Drugs and medical data was assessed at start and three-monthly for 24 months. Drugs were classified by anatomical-therapeutic-chemical code (ATC). Endpoints for outcome analysis were RFS and CSS in univariate (Kaplan–Meier curves and log-rank test, Cox regression for Hazard Ratio (HR)) and multivariate (Cox regression models) analyses. Of 113 patients, 52 had FD and 78 RC. Median age was 74 and 72 years, 83% and 82% were male. Drugs of 114 ATC classes were taken by 48 (92%) FD patients (median number 4.5/IQR 2–7.5) and 73 (94%) of RC patients (median 5/IQR 2–9). In univariate analysis (log-rank test (p)/Cox regression (HR, 95% CI, p)), polypharmacy (*p* = 0.036/HR = 2.83, 95% CI = 1.02–7.90, *p* = 0.047), calcium channel blockers (*p* = 0.046/HR = 2.47, 95% CI = 0.97–6.27, *p* = 0.057) and proton pump inhibitors (*p* = 0.015/HR = 3.16, 95% CI = 1.18–8.41, *p* = 0.022) had a significant negative impact on RFS in RC patients, statins (*p* = 0.025/HR = 0.14, 95% CI = 0.02–1.06, *p* = 0.057) a positive effect on RFS in FD patients, angiotensin-converting enzyme inhibitors (*p* = 0.008/HR = 10.74, 95% CI = 1.20–96.17, *p* = 0.034) and magnesium (*p* = 0.042/HR = 5.28, 95% CI = 0.88–31.59, *p* = 0.067) a negative impact on CSS in FD patients. In multivariate analysis, the only significant drug effects were the negative impact of angiotensin-converting enzyme inhibitors (HR = 15.20, 95% CI = 1.30–177.67, *p* = 0.030) and magnesium (HR = 22.87, 95% CI = 1.57–333.81), *p* = 0.022) on CSS in FD patients, and the positive impact of statins (HR = 0.12, 95% CI = 0.01–0.97, *p* = 0.047) on RFS in FD patients. Impact of non-oncologic drugs on RFS and CSS was small in this prospective study. Thus, appropriate treatment of comorbidities is encouraged.

## 1. Introduction

Urothelial carcinoma of the bladder (UCB) represents the tenth most common cancer worldwide and is characterized by high recurrence and mortality rate. In Europe, more than 204,000 new cases and 67,000 deaths occurred in 2020 [1]. Treatment recommendations depend on the characterization as non-muscle-invasive bladder cancer (NMIBC) or muscle-invasive disease (MIBC), tumour staging and grading [2]. Transurethral resection of the bladder (TUR-B) is mostly performed for NMIBC. For recurring high risk NMIBC and localized MIBC, radical cystectomy (RC) is the first-line therapy [2,3]. Unfortunately, recurrence rates are still high, e.g., about 70% for patients with localized disease and TUR-B and 30% for patients with RC [4].

UCB is about four times more prevalent in males than in females. For both sexes, the mean age at diagnosis is well over 70 years [3,4,5]. At this age, more than 90% of patients are on chronic drug therapy for pre-existing comorbidities like hypertension, hypercholesterolemia or diabetes mellitus [6,7,8]. Apart from well-known risk factors like tobacco smoking or occupational hazards, a growing body of evidence describes chronic diseases, like hypertension or metabolic syndrome, to be of concern for UCB development [5,9].

However, conflicting results have been published on the impact of non-oncologic chronically administered drugs on the risk of UCB development, recurrence and survival. For instance, a protective effect on cancer risks has been broadly discussed for statins and low-dose aspirin [10]. In contrast, a case–control study from Italy found no association between intake of aspirin or statins and UCB development [11].

Data on the influence of drugs targeting the renin–angiotensin–aldosterone– system (RAAS) are even more ambiguous. Regarding UCB development, a systematic review and meta-analysis found no association for angiotensin-converting enzyme inhibitors (ACEI), while angiotensin receptor blocker (ARB) slightly increased the risk [12]. Regarding recurrence and survival, small retrospective studies found improved outcomes for patients with NMIBC and MIBC taking ACEI or ARB [13,14,15,16]. However, in a retrospective evaluation of a higher number of patients from our hospital ACEI had no effect, while ARB intake negatively influenced CSS in univariate, but not multivariate analysis [17]. On the other hand, a retrospective cohort study from Finland found ARB use before UCB diagnosis was associated with slightly decreased risk of UCB death, as was post-diagnosis use, while ACEI had no effect [18]. Importantly, cardiovascular disease (CVD) was the main cause of death in older UCB patients according to a large retrospective population-based study [19]. Thus, adequate treatment of cardiovascular comorbidities is necessary while the impact of chronic non-oncologic drug therapy on recurrence and CSS is still not clear.

So far, almost all studies evaluating a possible drug impact on UCB recurrence and survival are of retrospective design. Retrospective assessment of drug history is bound to be error-prone, since patients do not remember previous drug treatment correctly, documentation of drug histories in clinical records is often poor, and health insurance data do not prove the actual intake of prescribed drugs and exclude self-medication. Prospective assessment of non-oncologic drugs taken by UCB patients should allow new insights into which drug classes are taken and how this might affect UCB recurrence and survival. Therefore, we conducted a prospective study including patients with a primary diagnosis of NMICB or scheduled RC due to UCB and documented a detailed drug history, changes in medication and tumour disease over time. The aims of our study were twofold. First, we wanted to prospectively assess how many drugs and which drug classes patients with a first diagnosis of UCB or RC are actually taking. Secondly, we wanted to evaluate a possible impact of non-oncologic drugs on recurrence-free survival (RFS) and cancer-specific survival (CSS).

## 2. Materials and Methods

A prospective, monocentric, observational study including patients with a first diagnosis (FD) of UCB and TUR-B or scheduled for RC due to UCB was started at the LMU University Hospital, Munich, a tertiary care hospital, in April 2018. Patients were eligible for inclusion when aged >18 years, agreed to participate and information on current medication was available. The recruitment phase was 24 months with an initial interview at hospital admission and follow up every three months over the next 24 months or until death. The study was conducted in accordance with the Declaration of Helsinki. Approval was obtained by the ethics committee of the LMU University Hospital, Munich (18-427). Patients had to sign informed consent of participation. The study is registered at the German Clinical Trial Register (DRKS00017080).

For study patients, the following data were assessed at hospital admission: age, sex, body weight and height; comorbidities (Charlson Comorbidity index (CCI), American Society of Anaesthesiologists Score (ASA), Eastern Cooperation Oncology Group index (ECOG)), UCB risk factors (occupation, smoking, alcohol consumption, illegal drugs), urologic symptoms (micro/macro haematuria, micturition disorder, pain) and tumour staging. Tumour pathological staging (tumour (T), lymph nodes (N), metastasis (M)), grading (G) and surgical margin (R) was undertaken according to TNM classification and World Health Organization grading criteria [3]. After a first diagnosis of BC, surveillance with cystoscopy was performed every three months. Intravesical instillation of Bacille Calmette–Guerin (BCG) was performed in high risk cases (e.g., high grade tumours). Indication for RC was muscle-invasive bladder cancer. For systemic chemotherapy, gemcitabine/cisplatin or atezolizumab were used.

In addition, a detailed personal interview was performed by a pharmacist on previous and current drug therapy. According to recommendations for the best possible drug history, a structured questionnaire was used and in addition to the interview any information on drug therapy documented in the hospitals electronic patient information system (SAP i.s.h.med, Cerner Corporation, North Kansas City, MO, USA) was collected [20]. In case of uncertainties, the patients’ practitioner or family members were contacted. Prescription and over-the-counter drugs, regular and on demand drug therapy, and information on patients´ compliance were documented. In detail, drug, dosage, start and, if a drug was stopped, end of therapy were documented.

Follow-up interviews were performed by telephone every three months and covered any changes in medication and results of in- or outpatient urological control examinations (ultrasound, cystoscopy, cytology, computer tomography, MRT, bone scintigraphy) and tumour staging. In case of death, cause of death as defined by treating physician or death certificate was documented. For multivariate analysis of FD patients, variables for the Sylvester Recurrence Score were assessed from patient charts and score calculated according to Sylvester et al. [21].

Drug therapy of patients was classified by the anatomical therapeutic chemical code (ATC) according to the WHO [22]. Qualitative (five-digit ATC) and quantitative (number of patients on drug) analysis was performed. Polypharmacy was defined as five drugs or more [23]. Drugs taken by at least ten patients were included in statistical analyses for RFS and CSS.

Qualitative variables are presented with their frequency distribution, quantitative variables as median and interquartile range. The number of drugs per patient was calculated; in cases of differences at several time points, the maximum number per patient was used. Endpoints for outcome analysis were RFS and CSS. Univariate outcome analysis regarding the influence of non-oncologic drugs taken by at least ten patients was performed using Kaplan–Meier curves, log-rank tests and the Cox regression for Hazard Ratio (HR) with a 95% confidence interval (95% CI). In addition, univariate analysis was performed for polypharmacy yes/no with Kaplan–Meier curve and log-rank test, and the number of drugs as continuous variable using Cox regression. Subsequently, for each cohort (first diagnosis, radical cystectomy) and each endpoint, multivariate analysis was carried out using Cox regression models with backward selection. Variables included age, gender, CCI, tumour stage and grade, and the univariately significant drugs for each setting. Age and CCI were considered as continuous variables, all other parameter as dichotomous. *p* values smaller than 0.05 were regarded as significant. Statistical analyses were performed using MedCalc Statistical Software version 22 (Ostend, Belgium).

## 3. Results

Overall, 113 patients agreed to participate and underwent the complete study protocol over 24 months follow-up. Of these, 52 had a first diagnosis of UCB and 78 a scheduled RC. Fifteen patients were included at the first diagnosis and later appeared again with RC. Patient characteristics are presented in Table 1. In both groups, the majority of patients was male (83% and 82%), median of the CCI was two (IQR 2–4) and tumour classification was mostly high grade. FD patients were slightly older (median age 74 years) than patients with RC (median age 72 years).

### 3.1. Non-oncologic Drugs Taken by UCB Patients

FD patients had a median number of 4.5 drugs (IQR 2–7.5), and 48 of the 52 patients (92%) took at least one drug. Polypharmacy was present in 26 cases (50%). Patients with RC had a median number of five drugs (IQR 2–9) and 73 of the 78 patients (94%) took at least one drug. Polypharmacy was present in 43 patients (55%).

Classification of patients´ non-oncologic drug therapy by five-digit ATC code revealed 114 different drugs/drug classes, including minerals, vitamins and homeopathic drugs. Table 2 displays the drug classes taken by at least ten patients. Drugs targeting the cardiovascular system (ATC class C) represented the most frequently used drug class, especially antihypertensives. Regarding all 113 included patients, 59 (52%) took RAAS inhibitors (30 ACEI, 29 ARB), 32 (28%) beta-blockers, 29 (25%) calcium-channel-blockers (CCB) and 23 (20%) thiazide diuretics. Every third patient (34; 30%) was on statin therapy. Drugs targeting blood coagulation were additionally common with 33 (29%) taking inhibitors of thrombocyte aggregation (acetylic salicylic acid (ASS) or clopidogrel) and 18 (16%) direct acting oral anticoagulants (DOAC). The most frequently reported drug was sodium bicarbonate, especially in patients with RC.

Drugs used for chemotherapy were BCG instillation in 11 cases, one patient with neo-adjuvant and two with palliative chemotherapy.

### 3.2. Univariate Analysis of the Influence of Non-oncologic Drugs on Survival of UCB Patients

For drugs taken by at least ten patients, univariate analysis for RFS and CSS was performed separately for FD and RC. Results are presented in Table 3 (log-rank test) and Supplementary Table S1 (Cox regression model).

For the 52 FD patients, 15 recurrences and five deaths were documented. The mean RFS in FD patients was 19.2 months. As shown in Table 3, intake of statins had a significant positive effect on RFS (*p* = 0.025). Regarding CSS, intake of ACEI (*p* = 0.008) and magnesium (*p* = 0.042) had a significant negative impact.

**Figure 1:**
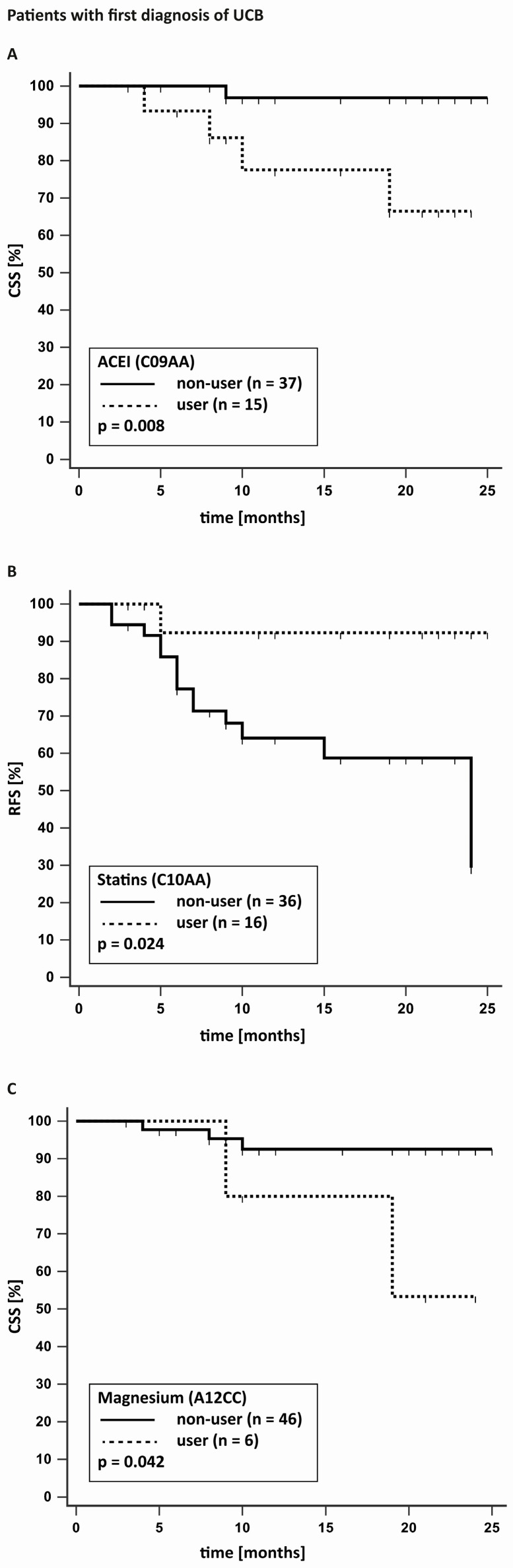
Kaplan–Meier curves for patients with a first diagnosis of urinary bladder cancer (UCB) with and without angiotensin-converting enzyme inhibitors (ACEI) (**A**) or magnesium (**C**) on cancer-specific survival (CSS) and statins (**B**) on recurrence-free survival (RFS).

**Figure 2:**
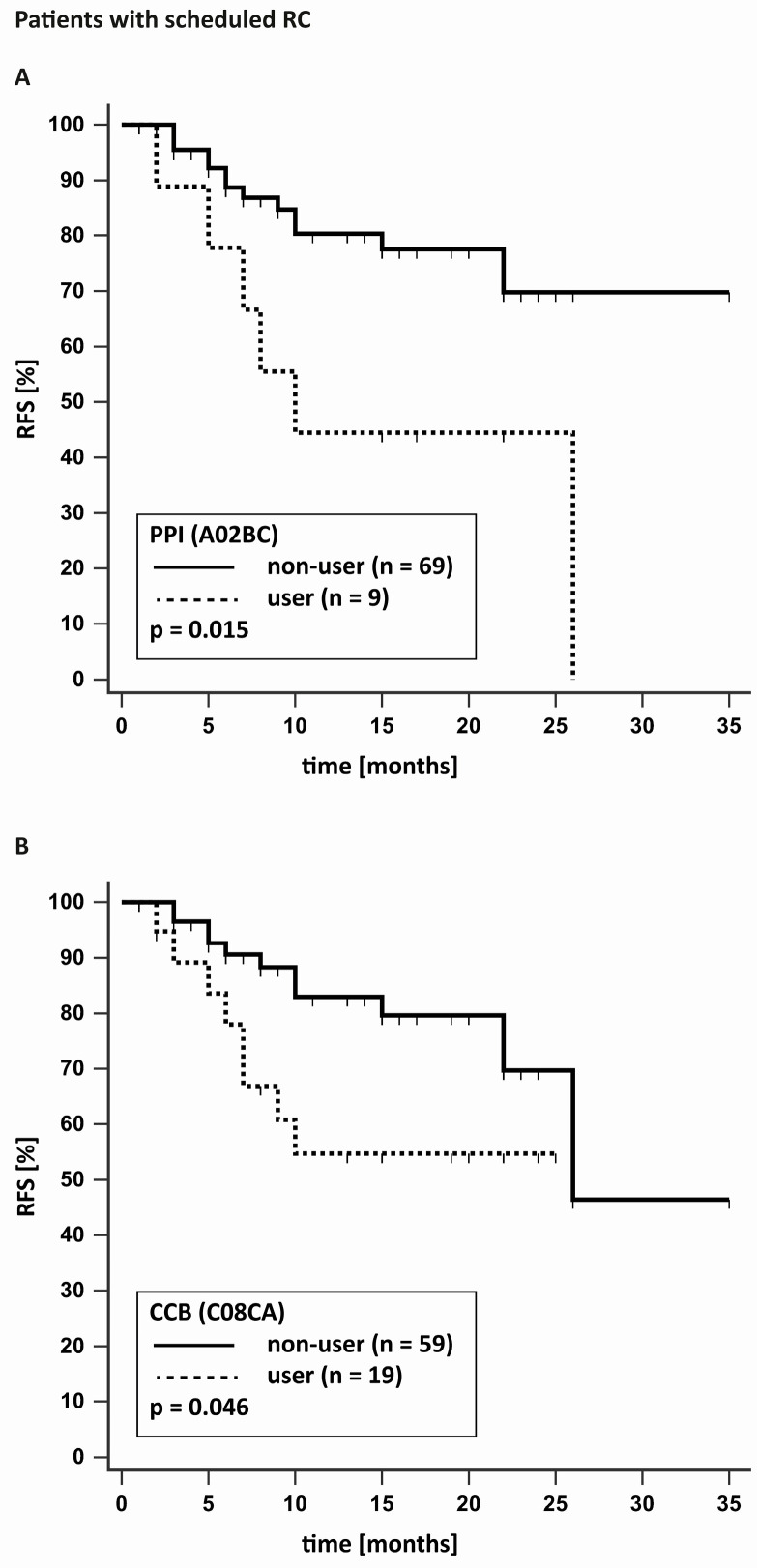
Kaplan–Meier curves for patients with scheduled radical cystectomy (RC) with and without proton-pump inhibitors (PPI) (**A**) or calcium-channel blockers (CCB) (**B**) for recurrence-free survival (RFS).

For the 78 patients with RC, 19 recurrences and 11 deaths were documented. The mean RFS was 24.7 months, with 21.8 months for low-grade and 17.2 months for high-grade tumours (*p* = 0.115). CSS was 25 months for low-grade and 21.4 months for high-grade tumours (*p* = 0.056). Intake of CCB (0.046) and proton pump inhibitor (PPI; 0.015) had a significant negative impact on RFS. Intake of sulfonamide diuretics achieved a *p* value of 0.05 exactly, with a possible negative impact on RFS. There was no significant impact of any drug on CSS. Figure 1 and Figure 2 present the Kaplan–Meier curves for the statistically significant results.

Univariate analysis using Cox regression instead of log rank tests revealed slightly different *p* values, as shown in Supplementary Table S1. In detail, for significant findings with the log-rank test, in Cox regression, the results were for calcium channel blockers HR = 2.47, 95% CI = 0.97–6.27 (*p* = 0.057), for proton pump inhibitors HR = 3.16, 95% CI = 1.18–8.41 (*p* = 0.022) on RFS in RC patients; for statins HR = 0.14, 95% CI = 0.02–1.06 (*p* = 0.057) on RFS in FD patients; for angiotensin-converting enzyme inhibitors HR = 10.74, 95% CI = 1.20–96.17 (*p* = 0.034) and magnesium HR = 5.28, 95% CI = 0.88–31.59 (*p* = 0.067) on CSS in FD patients.

Because of the possible impact of hypertension or antihypertensive drugs, a combined analysis regarding intake of any drug from ATC-classes C07AB, C09AA, C08CA, C09CA, C03AA and C03CA was undertaken (patients taking at least one of the drugs versus none). No statistically significant results were found for RFS (FD patients: *p* = 0.831; RC patients: *p* = 0.101) or CSS (FD Patients: *p* = 0.088; CSS patients: *p* = 0.220).

### 3.3. Univariate Analysis on the Impact of Number of Non-oncologic Drugs on Survival in UCB Patients

First, the presence of polypharmacy versus none was analysed using a log-rank test. In FD patients, no impact of polypharmacy was found on RFS (*p* = 0.548) and CSS (*p* = 0.118). In patients with RC, polypharmacy had a significant negative impact on RFS (*p* = 0.036), and univariate Cox regression showed a HR of 2.83 (95% CI 1.02–7.90; *p* = 0.047). No impact was found on CSS (*p* = 0.209) in RC patients.

Secondly, the number of drugs was tested as continuous variable. In FD patients, no effect was found for RFS (*p* = 0.691) and CSS (*p* = 0.062). In RC patients, no effect was found on CSS (*p* = 0.186), but on RFS (*p* = 0.043). Cox regression revealed a HR of 1.09 (95% CI 1.00–1.18) translating into an increased risk by 9% per drug taken.

### 3.4. Multivariate Analysis on the Impact of Non-oncologic Drugs and Additional Risk Factors on Survival in UCB Patients

For FD patients, multivariate analysis was performed for statins regarding RFS and for magnesium and ACEI regarding CSS. Co-variables were age, sex, CCI, pT ≥ 1, high-grade tumour and selected drug classes. As shown in Table 4, female gender and CCI increased the hazard ratio for RFS, and CCI as well on CSS. A possible protective effect of statins on RFS was significant, and intake of magnesium and ACEI had a significant negative impact on CSS.

For RC patients, multivariate analysis for RFS was performed for PPI and CCB intake with age, sex, CCI and tumour staging (pT3-4) as co-variables. Grading was not considered as a co-variable, since nearly all cases were rated as high grade. Results are presented in Table 4. In the multivariate analysis, no drug showed a statistically significant effect, and all drugs were subsequently removed in the backward analysis. Polypharmacy and the number of drugs had no impact on RFS in the multivariate analysis. CCI was the only independent prognostic factor for RFS in this study cohort followed for 24 months.

## 4. Discussion

To our knowledge, this is the first prospective study continuously assessing and analysing which drugs are taken by bladder cancer patients and the possible impact of non-oncologic drugs on recurrence and cancer-specific survival in detail. In our study cohort of 113 patients, more than 90% of the patients were on chronic non-oncologic drug therapy, with a median of four to five drugs per patient. Over 100 different drug classes categorized by ATC were taken by these patients. Antihypertensives, statins and drugs targeting blood coagulation represented the most frequently reported drug classes. Some drug classes showed a possible impact on RFS or CSS in the 24-month study period in the univariate analysis. In the multivariate analysis, however, only a significant negative impact on CSS was found for ACEI and magnesium in patients with a first diagnosis of UCB and a protective effect of statins. Most drug classes did not show an impact on RFS and CSS, and their use in UCB patients appears to be safe.

The median age in our study patients with bladder cancer was over 70 years, and more than 80% were male. Both results are in agreement with general findings for this oncologic entity [3,4,5]. Female gender proved to be a negative predictor for RFS in patients with a first diagnosis of UCB. This is in accordance with previous findings describing higher recurrence rate and mortality in women diagnosed with UCB compared to men [24,25]. The multivariate analysis revealed that CCI was associated with worse outcomes regarding recurrence in FD patients (HR 1.49) and RC (HR 1.54) and, in addition, had a negative impact on CSS (HR 2.06) in FD patients. In retrospective studies, a higher CCI was associated with various negative outcomes, e.g., more aggressive de novo vesical tumours or reduced survival after RC [26,27]. A systematic review on risk assessment tools for bladder cancer patients recommended the use of the CCI and its further evaluation in prospective studies, as performed in the study presented here [28].

We prospectively analysed for the first time how many and which non-oncologic drugs are taken by UCB patients continuously over a 24-month period. The only other prospective study concerning drugs in UCB patients we are aware of focused on lipid-lowering drugs only [29]. In contrast to retrospective studies focusing on drugs and UCB patients, where drug intake was mostly assessed at only one time point, we documented drug therapy every three months over a longer time period. Polypharmacy, usually defined as five or more drugs, was found in more than 50% of the patients. Similar results have been reported from other studies evaluating older cancer patients [30,31]. This presents a potential risk itself, since polypharmacy in cancer patients has been shown to increase the risk for drug interactions, drug toxicity, clinical events like falls, and to impact clinical outcomes [30,32]. For instance, in colorectal cancer patients aged over 65 years, polypharmacy was inversely associated with overall survival and CSS [33]. In our study, polypharmacy was a negative predictor for RFS in RC patients in the univariate analysis; otherwise, no effect was seen. Elderly cancer patients are at high risk for drug-related problems (DRPs), not only because of polypharmacy, but also due to altered organ functions and prescription of potentially inappropriate drugs (PIMs). DRPs have been found in up to 90% and PIMs in more than half of elderly cancer patients [30,34]. Therefore, since non-oncologic drugs are prescribed to almost all UCB patients, and many are on polypharmacy, DRPs are to be expected. Medication of these patients should be regularly evaluated to reduce preventable risks.

When looking at drugs taken by UCB patients in detail, sodium bicarbonate was the most frequent. It is commonly prescribed to patients with ileal neobladder construction and metabolic acidosis; accordingly, most patients were in the RC group [35]. Drugs targeting blood coagulation were common, in agreement with findings that CVD is the most frequent co-morbidity in UCB patients [11,36]. Interestingly, some patients were taking inhalative beta-2-agonists and glucocorticoids, indicating a concomitant asthmatic or chronic obstructive pulmonary disease.

Drugs targeting the cardiovascular system were the most frequently used drug classes in our study, especially antihypertensives with RAAS inhibitors (52% of all patients), beta-blockers (28%), CCB (25%) and thiazide diuretics (20%). This agrees with findings of a retrospective study describing hypertension as the most often reported co-morbidity in nearly 60% of all bladder cancer patients [36]. However, studies on the influence of hypertension or antihypertensives on UCB development, recurrence and survival have found conflicting results. For instance, in a retrospective study with more than 2000 patients, hypertension did not show an effect on development of bladder cancer, while an earlier meta-analysis found an increased risk for women, but not men [36,37]. In the prospective study presented here, the intake of ACEI had a negative impact on RFS in the univariate and multivariate analysis in patients with a first diagnosis of UCB, while ARB had no effect. Retrospective studies have found improved outcomes or no effect for ACEI and ARB in patients with UCB [13,14,15,16,17,18,38]. Several studies found different outcomes between use of ACEI and ARB [18,38].

CCB had a negative impact on RFS in the univariate, but not in the multivariate analysis in patients with RC in our study. Retrospective studies found no effect of CCB intake on RFS, CSS and OSS in UCB patients [14,17,18]. Interestingly, a recent study examining the associations of genetic proxies for CCBs with the risk for 17 site-specific cancers, including bladder cancer, found no correlation for any kind of cancer, thereby supporting the long-term safety profile of CCBs [39].

Beta-blockers did not show any influence on RFS or CSS in this prospective study. Similar findings have been published in retrospective and population-based evaluations [14,17,18]. In contrast, one register-based study from Sweden found beta-blocker use was associated with lower UCB-related mortality [40]. However, while in our study, beta-blocker use was assessed over the whole study period of 24 months, the Swedish study defined it as a prescription during 90 days prior to cancer diagnosis [40].

When considering the evidence from this prospective study and the literature reports so far, the impact of certain antihypertensive drug classes taken by UCB patients on recurrence and survival seems to be small. Presence of hypertension and CVD might be the more important factor. In a population-based study in older UCB patients (>65 years), death caused by CVD was the chief cause of death, even higher than UCB-related death and higher than in the general population of this age [19]. A retrospective study from the US including over 240,000 UCB patients confirmed CVD as leading cause for non-cancer death [41]. Recently, a positive association was confirmed between systolic blood pressure and the risk for aggressive UCB, especially in men with high genetic risk for bladder cancer [42]. Thus, appropriate treatment of hypertension and additional cardiovascular diseases should be the chief target, and the choice of drug seems to be of secondary importance. However, when analysing if the intake of any antihypertensive drugs had an impact on survival, no effect was seen in our study. Possibly, these effects will only be revealed in long-term studies.

Statins have repeatedly been discussed to have a positive impact on cancer recurrence and survival but, so far, data regarding UCBs are controversial. In our prospective study, 30% of the patients were on statin therapy, and a significant positive impact on RFS was found in univariate and multivariate analysis for a first diagnosis of UCB. No effect was seen on CSS after the first diagnosis of UCB, RFS and CSS in patients undergoing RC. The only other prospective study on this question analysed the influence of lipid-lowering drugs on the incidence of several cancer entities and mortality. In contrast to cancer of other origin, statin use was associated with a higher incidence of UCB; the effect was only seen in men [29]. A retrospective study on patients with NMIBC found higher-grade UCB at time of TUR-B and a lower risk of recurrence for statin use, but no influence on overall mortality [43]. Conflicting results have been found in additional retrospective studies reporting an increased odds ratio for developing UCB [44], no effect on recurrence and survival [17,25,45,46] or an increased recurrence rate [47]. Statins are prescribed to prevent atherosclerotic events, which are also dependent on other risk factors like smoking and metabolic disease. Differences in the presence of these risk factors in the study groups might explain the controversial study findings. However, in our opinion, there is no evidence to withhold statins in patients with UCB, since cardiovascular death plays such a major role in UCB patients.

PPI had a negative impact on RFS in univariate analysis, but not in multivariate analysis of patients with RC, otherwise no effect was seen. However, only nine patients undergoing RC reported taking PPI. We did not find other prospective studies focusing on the effects of PPI-intake on recurrence and survival in UCB patients. A retrospective case–control study from Scotland reported an increased risk for development of bladder cancer in patients using ranitidine, which is known to contain carcinogenic nitrosamines, but no effect for PPI [48]. Survival data concerning PPI intake in UCB patients focus on special treatments, like the effects of PPI on immune checkpoint inhibitors; a recent review found significantly worse outcomes in advanced-cancer patients regarding PFS and OSS [49]. Moreover, a recently published retrospective multicentre study evaluating patients with metastatic disease taking pembrolizumab found a significant negative impact of PPI intake in multivariate analysis on PFS and OS [46].

Surprisingly, magnesium intake had a negative impact on CSS in univariate and multivariate analysis in patients with a first diagnosis of UCB. However, only six patients reported intake of magnesium. Magnesium is often taken by patients as an over-the-counter drug for muscle cramps, constipation or generally as a lifestyle drug. The dosage taken can vary widely depending on the actual product. Only sparse clinical data could be found on a possible impact of magnesium or other minerals on UCB. Intake of vitamins and minerals, including magnesium, did not reduce the risk of UCB development in a study from the US [50]. However, the assessment of mineral and vitamin intake was only self-declared at the baseline. One study analysed serum levels of several trace elements and minerals in bladder cancer patients and found elevated levels of magnesium above reference ranges, but levels were comparable to a control group without UCB [51].

There are several limitations to our study. First, although we aimed for a complete drug history, we cannot rule out that some details are missing. However, drug history was taken by a trained pharmacist and according to recommendations for good clinical practice. In addition, we did not evaluate drug therapy regarding possible drug interactions, other DRP or PIM, since this was not the focus of our study. Future prospective studies should incorporate this issue. In addition, data on clinical follow-up were missing in some cases. Despite the high recurrence rate and mortality of UCB, the follow-up period of 24 months is possibly too short to discover some important effects of drugs on recurrence and survival and prevented analysis of overall survival. Some drug classes were taken only by few patients; thus, we limited statistical analyses to drugs taken by at least ten patients. Multicentre studies could help to gain higher patient numbers and enable to study a possible impact of rare drugs.

## 5. Conclusions

In a prospective study on UCB patients, we found that almost all patients were taking non-oncologic concomitant drug therapies, and many were on polypharmacy, which is associated with potential risks for DRP and worse treatment outcomes. A broad spectrum of drug classes was involved. There was only a small impact of certain drug classes on recurrence and cancer-specific survival in UCB patients. Drugs targeting the cardiovascular system were the most frequently identified drug class, and cardiovascular disease has been described as the main cause of death in UCB patients. Appropriate treatment of concomitant diseases is mandatory, while the choice of drug seems to be less important.

## Figures and Tables

**Table 1:** Patient characteristics.

Parameter		
**First Diagnosis Cohort (n = 52)**		
age [years]	median 74, IQR 63–79	
Sex		
male	83%	43
female	17%	9
Charlson comorbidity index	median 2, IQR 2–4	
Tumour classification		
pTa/is	62%	32
pT1	25%	13
>pT1	13%	7
Tumour grade		
low grade	37%	19
high grade	63%	33
**Radical cystectomy cohort (n = 78)**		
age [years]	median 72, IQR 63–78	
Gender		
male	82%	64
female	18%	14
Charlson comorbidity index	median 2, IQR 2–4	
Sylvester recurrence index	median 6, IQR 3–7	
Tumour classification		
pT0	17%	13
pTa/is	4%	3
pT1	15%	12
pT2	28%	22
pT3	6%	5
pT4	29%	23
Lymph node status		
pN0	81%	63
pN+	10%	8
pNX	9%	7
M0	99%	77
M1	1%	1
Tumour grade (only pT ≠ 0)		
low grade	5%	3
high grade	95%	62

**Table 2:** Drugs/drug classes taken by UCB patients classified by ATC code (intake by at least ten patients; n_tot_ = total; n_FD_ = first diagnosis; n_RC_ = radical cystectomy). ^a^ Sum of n_FD_ and n_RC_ is more than n_tot_, since some patients appeared in both groups.

ATC	n_tot_^a^ (of 114)	n_FD_ (of 52)	n_RC_ (of 78)	Drug
A02AH	42	10	41	Sodium bicarbonate
C10AA	34	16	19	Statins
B01AC	33	13	21	Antiplatelet drugs
C07AB	32	15	21	Beta-blockers
C09AA	30	15	22	Angiotensin-converting enzyme inhibitors
C08CA	29	12	19	Calcium channel blockers
C09CA	29	13	19	Angiotensin receptor blockers
C03AA	23	9	18	Thiazide diuretics
A11CC	23	12	13	Vitamin D
M04AA	21	7	14	Allopurinol/febuxostat
B01AF	18	10	10	Direct acting oral anticoagulants
C03CA	18	6	12	Sulfonamides (diuretics)
H03AA	17	9	12	Thyroid hormones
A02BC	16	8	9	Proton-pump-inhibitors
B03BA	14	3	11	Vitamin B12
G04CA	13	11	3	Alpha-blockers
R03AC	12	3	9	Inhalative beta-2-agonists
A12CC	12	6	8	Magnesium
R03BA	10	3	7	Inhalative glucocorticoides

**Table 3:** Univariate analysis with log-rank test on the impact of non-oncologic drugs on recurrence-free survival (RFS) and cancer-specific survival (CSS). Significant results are written in bold.

ATC	Drug	*p*-Value			
		RFS		CSS	
		First Diagnosis	RC	First Diagnosis	RC
A02AH	Sodium bicarbonate	0.930	0.253	0.943	0.173
C10AA	Statins	**0.025**	0.390	0.566	0.742
B01AC	Antiplatelet drugs	0.111	0.069	0.992	0.149
C07AB	Beta-blockers	0.540	0.099	0.151	0.183
C09AA	ACE inhibitors	0.665	0.082	**0.008**	0.294
C08CA	Calcium channel blockers	0.711	**0.046**	0.393	0.124
C09CA	Angiotensin receptor blockers	0.444	0.892	0.582	0.627
C03AA	Thiazide diuretics	0.895	0.051	0.183	0.450
A11CC	Vitamin D	0.856	0.269	0.333	0.635
M04AA	Allopurinol/febuxostat	0.456	0.574	0.471	0.767
B01AF	DOACs	0.517	0.480	0.328	0.368
C03CA	Sulfonamides	0.214	0.050	0.475	0.494
H03AA	Thyroid hormones	0.908	0.126	0.279	0.256
A02BC	Proton-pump-inhibitors	0.913	**0.015**	0.637	0.386
B03BA	Vitamin B12	0.408	0.774	0.616	0.298
G04CA	Alpha-blockers	0.539	0.115	0.783	0.062
R03AC	Inhalative beta-2-agonists	0.364	0.459	0.602	0.212
A12CC	Magnesium	0.084	0.289	**0.042**	0.412
R03BA	Inhalative glucocorticoides	0.364	0.743	0.602	0.282

**Table 4:** Multivariate analysis (Cox regression models with backward selection): recurrence-free survival (RFS) and cancer-specific survival (CSS) in first-diagnosis (FD) UCB patients and patients with radical cystectomy (RC).

Parameter	HR	95% Ci of HR	*p* Value
FD patients—RFS			
gender female	4.67	1.32–16.48	0.017
CCI	1.49	1.01–2.18	0.043
Statins	0.12	0.01–0.97	0.047
variables not included in the final model: age, pT ≥ 1, high grade			
FD patients—CSS			
CCI	2.06	1.08–3.95	0.029
Magnesium	22.87	1.57–333.81	0.022
ACEI	15.20	1.30–177.67	0.030
variables not included in the final model: age, gender, pT ≥ 1, high grade			
RC patients—RFS			
CCI	1.54	1.17–2.01	0.002
pT3-4	2.38	0.94–6.02	0.067
variables not included in the final model: age, gender, PPI, CCB			
RC patients—CSS			
no multivariate model (no significant drugs in univariate analysis)			

HR = hazard ratio; CI = confidence interval; RFS = recurrence-free survival; CSS = cancer-specific survival; CCI: Charlson Comorbidity Index; ACEI = angiotensin-converting enzyme inhibitors; PPI = proton pump inhibitors; CCB = calcium-channel-blocker.

## Data Availability

Data are available upon reasonable request.

## Supplementary Materials

The following supporting information can be downloaded at: https://www.mdpi.com/article/10.3390/jcm12216749/s1, Table S1: Univariate analysis (Cox regression model) of the impact of drugs on recurrence-free survival (RFS) and cancer-specific survival (CSS).
