# Supplementary material for: The Characterization of Non-oncologic Chronic Drug Therapy in Bladder Cancer Patients and the Impact on Recurrence-Free and Cancer-Specific Survival: A Prospective Study
Source: J Clin Med. 2023 Oct 25;12(21):6749. doi: 10.3390/jcm12216749 (PMC10648271; doi:10.3390/jcm12216749)
Supplement: Supplementary file 1 [file jcm-12-06749-s001.zip › jcm-2639454-supplementary.pdf]

Table S1. Univariate analysis (Cox regression model) on the impact of drugs on recurrence free survival (RFS) and cancer specific survival (CSS).

**Drug**	**RFS**			**CSS**		
	**HR**	**95% CI**	**p value**	**HR**	**95% CI**	**p value**
**First diagnosis cohort**						
Sodium bicarbonate	1.06	0.29 – 3.80	0.931	1.08	0.12 – 9.75	0.944
Statins	0.14	0.02 – 1.06	0.057	0.53	0.06 – 4.77	0.572
Antiplatelet drugs	0.23	0.03 – 1.72	0.150	1.00	0.11 – 9.06	0.998
Beta-blockers	1.40	0.47 – 4.19	0.547	3.44	0.57 – 20.70	0.177
ACE-inhibitors	1.26	0.43 – 3.70	0.670	10.74	1.20 – 96.17	0.034
Calcium-channel-blockers	0.79	0.22 – 2.80	0.715	2.14	0.36 – 12.83	0.405
Angiotensin-receptor-blockers	0.62	0.17 – 2.19	0.454	0.54	0.06 – 4.91	0.587
Thiazide diuretics	1.09	0.31 – 7.87	0.897	3.17	0.53 – 19.15	0.208
Vitamin D	0.89	0.25 – 3.16	0.858	2.36	0.39 – 14.11	0.348
Allopurinol/ febuxostat	0.47	0.06 – 3.62	0.472	2.20	0.24 – 19.78	0.482
DOACs	1.45	0.46 – 4.56	0.525	2.38	0.40 – 14.28	0.343
Sulfonamides	2.29	0.60 – 8.79	0.228	2.18	0.24 – 19.61	0.487
Thyroid hormones	1.08	0.30 – 3.82	0.910	0.04	0.00 – 556.42	0.499
Proton-pump-inhibitors	0.92	0.21 – 4.10	0.915	1.69	0.19 – 15.19	0.641
Vitamin B12	2.29	0.30 – 17.73	0.427	0.05	0.00 – 3.7 x 10^6	0.741
Alpha-blockers	0.67	0.19 – 2.43	0.546	0.74	0.08 – 6.63	0.784
inhalative beta-2-agonists	0.05	0.00 – 1322.33	0.556	0.05	0.00 – 2.1 x 10^6	0.732
Magnesium	2.90	0.80 – 10.46	0.104	5.28	0.88 – 31.59	0.069
inhalative glucocorticoides	0.05	0.00 – 1322.33	0.556	0.05	0.0 – 2.1 x 10^6	0.732
**Radical cystectomy cohort**						
Sodium bicarbonate	0.58	0.23 – 1.50	0.264	0.42	0.11 – 1.61	0.208
Statins	1.52	0.57 – 4.03	0.399	1.23	0.33 – 4.55	0.757
Antiplatelet drugs	2.26	0.91 – 5.64	0.080	2.23	0.68 – 7.35	0.185
Beta-blockers	2.11	0.84 – 5.30	0.110	2.11	0.64 – 6.49	0.220
ACE-inhibitors	2.19	0.88 – 5.48	0.093	1.99	0.53 – 7.40	0.306
Calcium-channel-blockers	2.47	0.97 – 6.27	0.057	2.44	0.71 – 8.37	0.157
Angiotensin-receptor-blockers	1.07	0.38 – 3.02	0.894	1.41	0.35 – 5.63	0.631
Thiazide diuretics	2.40	0.96 – 5.97	0.061	1.56	0.45 – 5.36	0.480
Vitamin D	1.77	0.63 – 4.95	0.280	1.35	0.35 – 5.19	0.659
Allopurinol/ febuxostat	0.66	0.15 – 2.88	0.581	1.27	0.26 – 6.11	0.769
DOACs	1.55	0.45 – 5.38	0.489	2.02	0.42 – 9.77	0.381
Sulfonamides	2.55	0.96 – 6.77	0.061	1.54	0.40 – 5.94	0.529
Thyroid hormones	2.18	0.78 – 6.14	0.139	0.04	0.00 – 237.44	0.469
Proton-pump-inhibitors	3.16	1.18 – 8.41	0.022	1.97	0.41 – 9.50	0.398
Vitamin B12	0.84	0.24 – 2.89	0.777	0.37	0.05 – 2.96	0.351
Alpha-blockers	4.45	0.57 – 34.65	0.154	5.97	0.70 – 51.20	0.103
inhalative beta-2-agonists	0.58	0.13 – 2.55	0.468	0.04	0.00 – 124.35	0.430
Magnesium	1.81	0.59 – 5.52	0.300	1.91	0.39 – 9.26	0.423
inhalative glucocorticoides	0.78	0.18 – 3.48	0.745	0.04	0.00 – 358.93	0.491

HR = hazard ratio; CI = confidence interval.
